# The gut: a triggering place for autism – possibilities and challenges

**DOI:** 10.3402/mehd.v23i0.18982

**Published:** 2012-08-24

**Authors:** Tore Midtvedt

**Affiliations:** Department of Microbiology, Tumor and Cell Biology (MTC), Karolinska Institutet, Stockholm, Sweden

**Keywords:** autism, intestine, microbiota, *Clostridium*, *Desulfovibrio*, *Sutterella*

## Abstract

**Background:**

During the recent years, a substantial amount of new data has underlined the importance of the gut as a triggering place for autism. Temporary improvements in clinical status following dietary alterations and the same that may occur after an antibiotic therapy are reported. Additionally, increasing numbers of bacteria belonging to certain groups, such as clostridia, desulfovibrios, and sutterella, have been reported. So far, however, presence of any bacterial group has never been causatively linked to autism, and every time a new candidate organism is introduced the same questions have to be asked: What is the cause? What is the consequence? What is the confounder? The possibilities of answering these questions are hampered by difficulties in obtaining adequate samples. Therefore, more efforts have been made to those biochemical methods that probe possible functional alterations in the gastrointestinal (GI) microbiota in autistic children.

**Conclusion:**

Autism is a disorder involving many organs and their functions, including the GI microbiota. More knowledge about the GI microbiota and its cross-talks with the host creates possibilities for future diagnostic and therapeutic improvements.

During the several decades after the first description of autism (1) in the early 1940s, autism was looked upon as a rare, non-treatable, degenerative disorder of the brain of unknown etiology. Most attention was paid to characterize the behavioral and genetic aspects in the child and psycho-sociological aspects in the family of the child.

However, an increasing number of reports, mostly from parents, of temporary improvements following dietary alterations and/or exposure – for other reasons – to antibiotics started to appear in the 1990s, and these reports served as an eye-opener for a broader view on the etiological and genetic aspects of autism and more systematic, therapeutic approaches (2). Very soon, alteration in the composition of the GI microbiota in autistic children compared with controls started to appear.

In the initial studies, attempts were made to define and isolate the possible, specific intestinal bacterial species responsible for the disorder. Various species in the clostridial groups, such as *Clostridium tetani*, *Clostridium perfringens*, *Clostridium bolteae* novo sp., etc. (3, 5), were reported to occur more frequently in the fecal samples of autistic children than in those of controls. However, despite a substantial amount of efforts, it has not been possible to clarify whether these findings represent the presence of a causative agent, reflect a consequence of previous treatment of the child, or are nothing but confounders. Recently, the focus has been on the presence of two other genera, *Desulfovibrio* (6) and *Sutterella* (7, 8). Again, it has not been possible to establish any causative relationship between the presence of any of these groups and the development of autism.

It is now more and more realized that the development of autism is not caused by the presence/absence of one – or a few – bacterial species, but may be caused by functional alterations of the GI microbiota. In order to evaluate such possible alterations, it has to be kept in mind that the GI tract consists of several functional compartments, each harboring its own microbiota. Biochemical reactions – either microbial- or host-derived – are governed by the ‘microclimate’ in the respective compartment. Out of the many factors that may influence the microclimate, pH, reduction/oxidation (red/ox) potential, and oxygen tension are of imperative importance. The values shown in Figs. 1–3 are based on studies conducted in healthy adults. So far, pH is the easiest parameter to follow. Recent improvements in technology allow us now to investigate pH individually in the whole GI tract collectively. (Fig. 1). Values regarding red/ox potential (Fig. 2) and oxygen tensions (Fig. 3) are hampered by a considerable degree of uncertainty because of technical problems of *in vivo* measurements. The values shown in Fig. 2 are related to human content, whereas the values shown in Fig. 3 reflect oxygen tension on luminal cell surfaces. Oxygen tensions in luminal content are assumed to be close to zero in the lower part of ileum and in the colon.

**Fig. 1:**
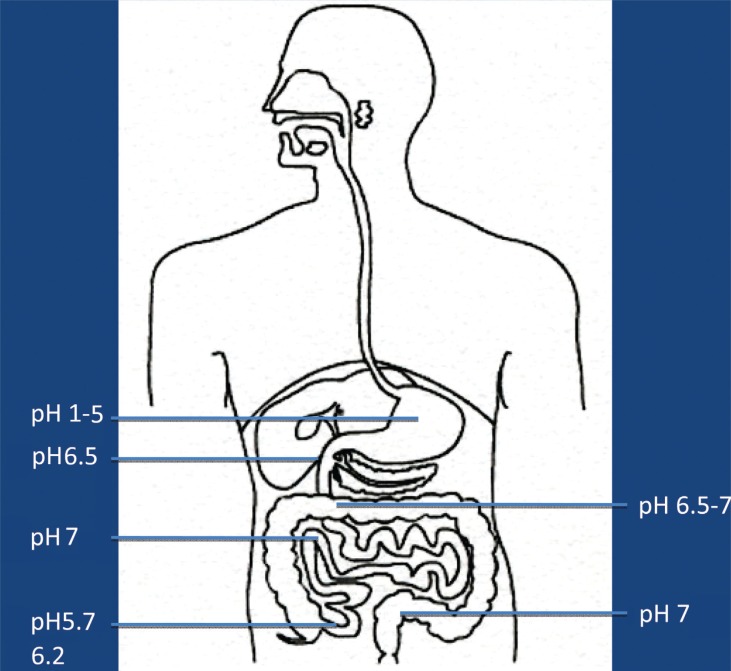
Main values of pH in luminal content in healthy adults.

**Fig. 2:**
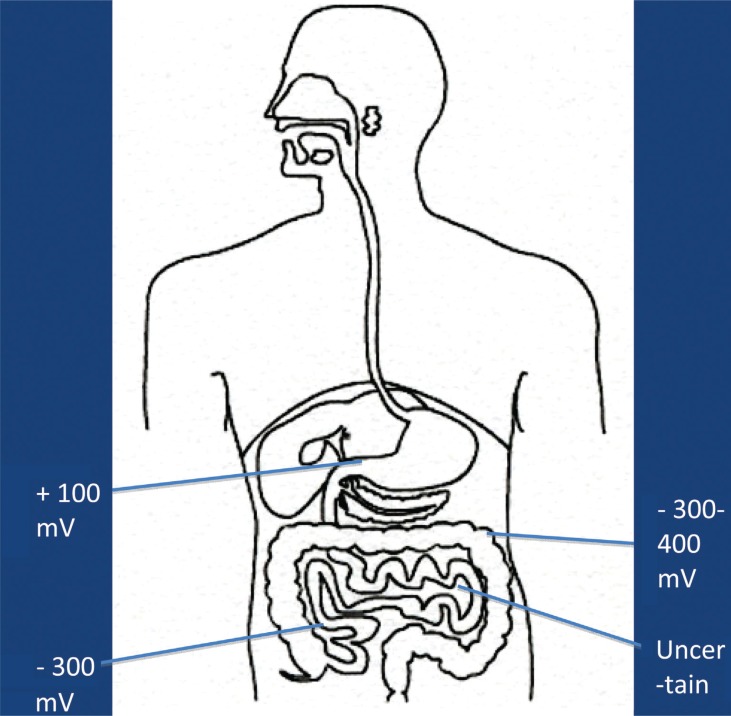
Main values of red/ox potential in luminal content in healthy adults.

The fate of any compound – dietary or environmentally derived – reaching any of these compartments will depend on the composition of the microbiota and the microclimate in that specific compartment. It should also be mentioned that neither the composition nor the microclimate is a static parameter; rather they are influenced by a wide variety of factors, such as diet, antibiotics, age, etc. Additionally, except for measurements of pH, it is at present not possible to follow alterations in red/ox potential, oxygen tension, or the composition of the microbiota in most of these compartments. Therefore, we have to rely on the evaluation of fecal microbiota and on products, derived from biochemical reactions, present in feces, urine, or serum, reflecting microbial/host metabolism of endogenous and exogenous compounds.

**Fig. 3:**
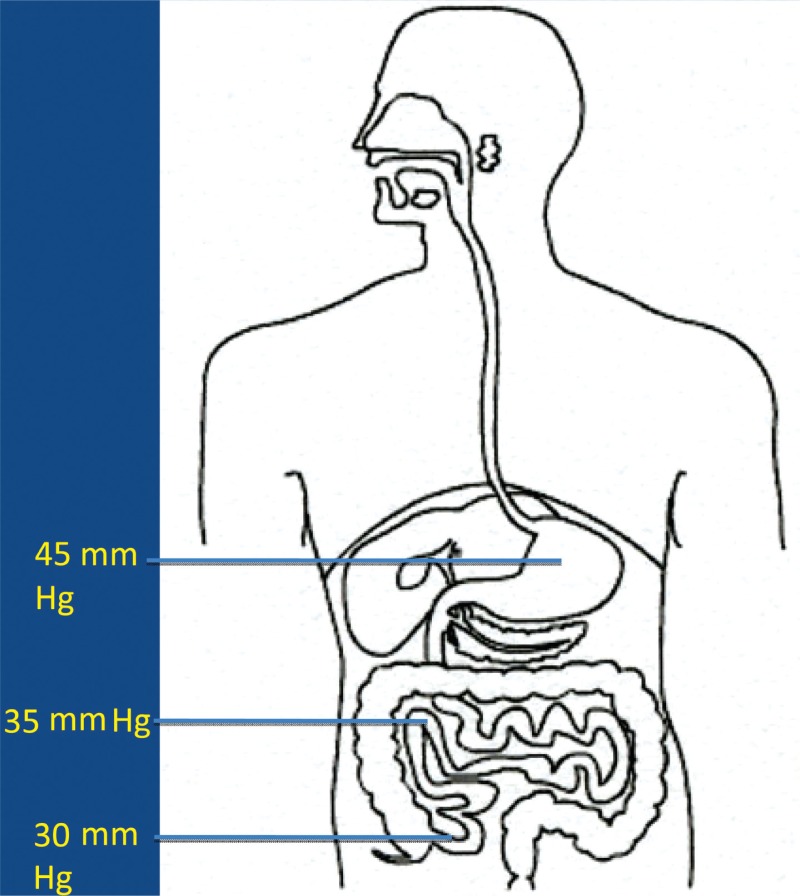
Oxygen tension on luminal cell surfaces in healthy adults.

It may sound difficult – and it is. Out of the many attempts that have been made to evaluate the possible microbial-derived biochemical alterations, I will briefly comment on three main approaches.

1. Alteration in sulfur metabolism: It has been observed that autistic children have a greater urinary excretion of sulfur than the controls. The GI tract is by far the most sulfur-rich organ in the body. Sulfur is a substantial part of the huge amounts of mucus produced daily in the GI tract, and several sulfur-containing compounds (sulfate, taurin, etc.) are excreted as conjugates of bile. Parts of the GI microbiota may produce hydrogen sulfide –a compound not only more toxic than cyanide, but also a well-known neurotransmitter. It is also known that the degree of mucus sulfation and the presence of sulfate-containing bile conjugates are altered during infancy. Whether, and to what extent, such alterations can be found in autistic infants have, to the best of my knowledge, not been evaluated.
2. Production of some organic acids, especially propionic acid (9): Short chain fatty acids (acetic, propionic, butyric, valeric, and caproic acids) represent microbial anaerobic degradation of carbohydrates. Most of these acids are absorbed and may influence many functions in the human body. It has now been convincingly shown in animal models that propionic acid induces behavioral and functional alterations in the brain of exposed animals. Most likely, the presence of this acid alters basic mitochondrial functions, thus underlining the importance of alterations in mitochondrial functions in autistic children, hypothesized already in the late 1990s (10).
3. The presence of bioactive peptides in urine (11): It is a well-known fact that many autistic infants improve on a casein- and gluten-free diet. Biochemical analysis of urine samples collected from children with autism shows an altered urinary peptide profile compared with those from the age-matched controls, and that this discrepancy decreased significantly after changing to a casein- and gluten-free diet in the cohort of children with autism. Additionally, there are several reports describing peculiar peptides in urine and/or serum from autistic children (12, 13) and animal models (14). Taken together, these reports support a view that the host/microbe metabolism of protein is altered in patients with autism. However, the mechanism(s) behind and the specific use of such alterations as biomarkers in diagnosing autism is (are) not sufficiently evaluated.

Since autism was first described in the 1940s, it has been looked upon as a rare disorder, occurring at a rate of 4–5 per 10,000 children. In the 1970s, the incidences started to rise and have continued to do so in nearly all countries reported (15). Now autism is generally accepted to be a grand challenge to global mental health. Data from the Unites States in 2010 indicate an incidence rate of 1/188 and data from South Korea indicate an incidence as high as 1/38. The mechanism(s) behind this dramatic increase is of course a matter of great concern (16). A genetic aspect is well accepted in some cases of autism. The key question is whether this increased incidence is genotypically or phenotypically based, i.e. reflects an increased mutation rate (chromosomal or mitochondrial) induced by factors not yet identified or is caused by environmental factors acting on already existing phenotypes. The geographical variations, especially the extremely high rate of incidences reported from South Korea, indicate that the latter possibility might be the most plausible one. A glance through the historical window might strengthen this assumption. Clioquinol was introduced in the 1930s as an amebicide drug, but was soon used worldwide as an anti-diarrheal drug – although its use was questioned very early (17). In the 1960s a peculiar neurological disease became prevalent in the Far East, especially in Japan. It was named subacute myelo-optic neuropathy (SMON), characterized by sensory and motor disturbances and visual changes, often leading to blindness. After some 10,000 cases worldwide, but especially in the Far East, including Japan, SMON was associated to the use of clioquinol, and the drug was withdrawn from the market (18). It has been known for years that there is an ethnic peculiarity in some mitochondrial function in people from Far East, and this has been assumed to be involved in the high incidence of SMON in Japan. It is now generally accepted that clioquinol is a very potent zinc and copper chelator, and that it may – under specific circumstances – act as a potent mitochondrial toxin (18, 19). As for a personal speculation, initially it was the presence of green urine in some patients with SMON that triggered some chemical analyses, thereby unmasking the fact that ‘this disease was caused by the intoxication of the administered Clioquinol’ (20). Green urine is sometimes found in autistic children. To the best of my knowledge, neither the mechanism(s) behind nor the presence of possible toxicant(s) has been satisfactorily evaluated.

The burning question in autism research today is: how to unmask the patho-physiological mechanisms behind the development of autism in a child? (21). It is more and more realized that a major key in this aspect is increased knowledge of the many complicated biochemical interactions established between a child and its GI microbiota in the first few years of its life, i.e. the period of life for autism to occur. A way forward might be to closely follow a large cohort of newborns – microbiologically as well as biochemically – from birth up to 2–3 years of age, in order to unmask the possible early deviations in these interactions in individuals, later developing autism. It is widely acknowledged that early identification and intervention is of imperative importance in several disorders included in the group of ASD (especially phenylketonuria and Rett syndrome (22) and the same may hold true also for autism). Additionally, the mere fact that dietary interventions change behavior in an autistic child (2) clearly underlines the functional, more than the degenerative, aspect in the pathogenesis of autism. The world-wide rapid increase in the incidence of autism is a challenge, as it indicates that it is human-made, and consequently it has to be solved by humans.
